# Successes and lessons learned in database development for national multi-site cancer care delivery research trials: the Alliance for Clinical Trials in Oncology experience

**DOI:** 10.1186/s13063-022-06536-x

**Published:** 2022-08-09

**Authors:** David Zahrieh, Shauna L. Hillman, Angelina D. Tan, Jennifer L. Frank, Travis Dockter, Bobbi Jo Meyers, Cassie L. Cherevko, Elizabeth S. Peil, Shaylene McCue, Oudom Kour, Heather J. Gunn, Heather B. Neuman, George J. Chang, Electra D. Paskett, Sumithra J. Mandrekar, Amylou C. Dueck

**Affiliations:** 1 Department of Quantitative Health Sciences and Alliance Statistics and Data Management Center, Mayo Clinic, Rochester, MN 55905 USA; 2 Department of Surgery, Division of Surgical Oncology, University of Wisconsin, School of Medicine and Public Health, Madison, WI USA; 3 Department of Colon and Rectal Surgery, Division of Surgery, The University of Texas MD Anderson Cancer Center, Houston, TX USA; 4 Department of Medicine, College of Medicine, Comprehensive Cancer Center, The Ohio State University, Columbus, OH USA

**Keywords:** Cancer care delivery research, Clinical trial management and optimization, Clinical trial operations, Data management and monitoring

## Abstract

**Introduction:**

Alliance for Clinical Trials in Oncology (Alliance) coordinated trials utilize Medidata Rave® (Rave) as the primary clinical data capture system. A growing number of innovative and complex cancer care delivery research (CCDR) trials are being conducted within the Alliance with the aims of studying and improving cancer-related care. Because these trials encompass patients, providers, practices, and their interactions, a defining characteristic of CCDR trials is multilevel data collection in pragmatic settings. Consequently, CCDR trials necessitated innovative strategies for database development, centralized data management, and data monitoring in the presence of these real-world multilevel relationships. Having real trial experience in working with community and academic centers, and having recently implemented five CCDR trials in Rave, we are committed to sharing our strategies and lessons learned in implementing such pragmatic trials in oncology.

**Methods:**

Five Alliance CCDR trials are used to describe our approach to analyzing the database development needs and the novel strategies applied to overcome the unanticipated challenges we encountered. The strategies applied are organized into 3 categories: multilevel (clinic, clinic stakeholder, patient) enrollment, multilevel quantitative and qualitative data capture, including nontraditional data capture mechanisms being applied, and multilevel data monitoring.

**Results:**

A notable lesson learned in each category was (1) to seek long-term solutions when developing the functionality to push patient and non-patient enrollments to their respective Rave study database that affords flexibility if new participant types are later added; (2) to be open to different data collection modalities, particularly if such modalities remove barriers to participation, recognizing that additional resources are needed to develop the infrastructure to exchange data between that modality and Rave; and (3) to facilitate multilevel data monitoring, orient site coordinators to the their trial’s multiple study databases, each corresponding to a level in the hierarchy, and remind them to establish the link between patient and non-patient participants in the site-facing NCI web-based enrollment system.

**Conclusion:**

Although the challenges due to multilevel data collection in pragmatic settings were surmountable, our shared experience can inform and foster collaborations to collectively build on our past successes and improve on our past failures to address the gaps.

**Supplementary Information:**

The online version contains supplementary material available at 10.1186/s13063-022-06536-x.

## Introduction

To generate generalizable knowledge that can lead to evidence-based practice change, high-quality multidisciplinary, multisite interventional and observational cancer care delivery research (CCDR) trials are conducted [1, 2]. Because the scope of CCDR encompasses patients, providers, practices, and their interactions, a defining characteristic of CCDR trials is hierarchical or multilevel data collection in real-world settings. In contrast to cancer therapeutic trials, which collect patient-level efficacy and safety data within the confines of a well-controlled setting, CCDR trials collect data on patients, providers, and participating clinics in pragmatic settings with the aim to improve delivery of cancer care in community and academic practices.

In accordance with directives from the National Cancer Institute (NCI), the Alliance for Clinical Trials in Oncology (Alliance) NCI Community Oncology Research Program Research Base (Alliance NCORP) CCDR Committee has worked to position the Alliance NCORP as a national leader in CCDR. This preparatory work has charted the future directions of CCDR within the Alliance NCORP, led to collaborations with the SWOG [3] and the Children’s Oncology Group (COG) research [4], and yielded a robust trial portfolio. Our portfolio comprises five trials (most with behavioral interventions), all of which capture multilevel data, both quantitative and qualitative, in pragmatic settings. Each is inherently complex and differs from conventional cancer therapeutic trials. To test the effectiveness of interventions as applied in the real world rather than under ideal conditions, the designs consist of a parallel-arms cluster randomized trial (CRT), a 2-by-2 factorial CRT, stepped-wedge designs, including a stepped-wedge design that implements randomization at two levels (patient- and practice-level), and an observational study. Because CCDR provides an opportunity to examine how clinician and health-system factors affect equity in cancer care for racial/ethnic groups and medically underserved populations, these trials often target minority or underserved patients.

The Statistics and Data Management Center (SDMC) at the Mayo Clinic Cancer Center (MCCC) has a rich history in coordinating multisite clinical trials, starting with serving as the SDMC for the North Central Cancer Treatment Group (1977-2012), and now part of the Alliance. All Alliance trials utilize Medidata Rave® (Rave) as the primary electronic data capture system. Our experience in conducting clinical trials notwithstanding, the CCDR trials required innovative strategies to address unanticipated challenges that arose with database development, centralized data management, and data monitoring in pragmatic settings. Because CCDR research is largely in its infancy, there is a dearth of external experience in conducting such trials to provide guidance.

Having recently implemented five CCDR trials utilizing the Alliance system infrastructure and Rave, our objective was to share our approach to analyzing the database development needs of the included trials and to describe the novel strategies applied to overcome the unanticipated challenges we encountered. The innovative strategies are organized into 3 categories: multilevel (clinic, clinic stakeholder, patient) enrollment; multilevel quantitative and qualitative data capture, including nontraditional data capture mechanisms such as Qualtrics surveys and call centers; and multilevel data monitoring. We conclude with a discussion on several remaining points of consideration and gaps that may need to be addressed when developing and implementing CCDR trials.

## Clinical trials

Five Alliance NCORP CCDR trials (A191402CD, A231601CD, A231602CD, A231701CD, A231901CD) are used to illustrate the unanticipated challenges associated with database development, centralized data management, and data monitoring. A tabular description of each trial is shown in Table 1.

**Table 1:** The alliance NCORP CCDR portfolio

Alliance #	Title	CT.gov Identifier	Study Design	Multilevel Data Collection			Quantitative Data		Qualitative Data	
				Levels	BL	FWP	Yes/No	Mechanism	Yes/No	Mechanism
A191402CD	*Testing decision aids to improve prostate cancer decisions for minority men*	NCT03103321	2x2 factorial cluster randomized trial	Clinics	Yes	No	Yes	Paper	No	-
				Patients	Yes	Yes	Yes	Paper	No	-
A231601CD	*Improving surgical care and outcomes in older cancer patients through implementation of an efficient presurgical toolkit (OPTI-Surg)*	NCT03857620	Parallel arms, cluster randomized trial	Clinics	Yes	Yes	Yes	Paper	No	-
				Providers and non-providers	Yes	Yes	Yes	Paper	Yes	Clinic observation / interviews
				Patients	Yes	Yes	Yes	Paper	No	-
A231602CD	*Assessing financial difficulty in patients with blood cancers*	NCT03870633	Observational	Clinics	Yes	No	Yes	Paper	No	-
				Patients	Yes	No	Yes	Telephone survey	No	-
A231701CD	*Increasing socioeconomically disadvantaged patients’ engagement in breast cancer surgery decision making through a shared decision-making intervention*	NCT03766009	Stepped-wedge	Clinics	Yes	No	Yes	Paper	No	Fields notes from observations
				Surgeons	Yes	No	Yes	Paper	Yes	Interviews
				Patients	Yes	Yes	Yes	Paper and Qualtrics survey; audio-recordings of clinic consults	Yes	Interviews/Focus groups
A231901CD	*Improving Patient-Centered* *Communication in Breast Cancer: A RCT of a Shared Decision Engagement System (ShaRES)*	NCT04549571	Stepped-wedge, including patientlevel randomization within a wave	Clinics	Yes	No	Yes	Paper	No	-
				Clinicians^b^	Yes	Yes	Yes	Paper	Yes	Interviews
				Patients	Yes	Yes	Yes	ePRO^a^	Yes	Interviews

*BL* Baseline, *FWP* Follow-up occasions.

BL data collection can occur at the time of site-level activation (e.g. baseline clinic surveys) or at the time of patient enrollment (e.g. baseline patient surveys).

A “Provider” is someone who has licensed prescriptive authority and includes Medical Doctor/Doctor of Osteopathy (MD/DO), Physician’s Assistant (PA), and Nurse Practitioner (NP).

^a^Electronic patient-reported outcome data capture system.

^b^Clinicians include the participating surgeons and their designees (providers such as physician assistants and nurse practitioners; and other professional disciplines such as registered nurses and social workers).

## Unanticipated challenges and strategies

### Multilevel enrollment

The enrollment of patients, clinic stakeholders (providers, who have prescriptive authority, and other professional disciplines such as registered nurses), and clinics requires that we have separate Rave study databases to capture the level-specific data such that the data are linked across the hierarchy and that the study interventions were correctly assigned. When we began to conduct CCDR studies, the challenges with multilevel enrollment were threefold. First, clinics and consented clinic stakeholders could not initially be enrolled in the site-facing NCI web-based enrollment system Oncology Patient Enrollment Network (OPEN); OPEN captures trial enrollments onto NCI-sponsored Network Group clinical trials and is integrated with the Cancer Trials Support Unit (CTSU) Enterprise System for regulatory and roster data, and with each of the Network groups’ registration/randomization systems to facilitate registration and randomization. Second, after OPEN was updated in 2020 to include clinic- and clinic stakeholder-enrollment, complementary functionality within the Alliance SDMC is needed to be built to take advantage of this new multilevel enrollment feature in OPEN. Third, a study with patient-level interventions that needed to be delivered centrally by the study team (rather than by the enrolling site), as with A231901CD, required that the patient-level randomization assignment generated by the Research Enrollment Application be accessible to the study team immediately after enrollment so that the correct intervention could be sent to the patient within 24 h of enrollment.

**Fig. 1:**
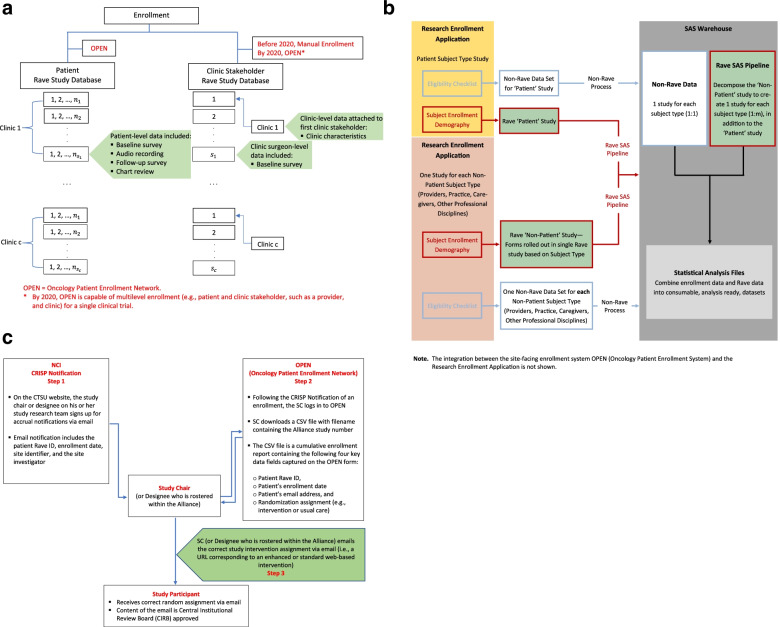
**a** Multilevel enrollment: study A231701CD. **a** Presents the hierarchical enrollment to study A231701CD prior to the OPEN enhancement that permitted multilevel enrollment. Patients are nested within clinic surgeons, who are nested within clinics. Each clinic enrolled patients, and the patients were enrolled in OPEN. The study also enrolled the clinic and clinic surgeons with manual enrollment, attaching clinic-level data with the first surgeon enrolled at the clinic. Here we assume c clinics such that clinic stakeholders are at Clinic 1, clinic stakeholders are at Clinic 2, and so on up to clinic stakeholders at Clinic c. Patients are nested within clinic stakeholders such that the first clinic stakeholder at Clinic 1 sees 1, 2, …, patients, the second clinic stakeholder at Clinic 1 sees 1, 2, …, patients, and so on. **b** Summary of data flow between the research enrollment application, rave, and statistical analysis files. **c** Distributing the randomly assigned study intervention

Figure 1a illustrates our initial strategy to hierarchical enrollment for Alliance NCORP CCDR study A231701CD. Prior to NCI’s recent update to OPEN to permit hierarchical enrollment, clinic stakeholders (e.g., a patient’s surgeon) were enrolled by the Alliance Registration Office; stated differently, a paper enrollment form for the clinic stakeholder was completed, emailed to the Alliance Registration Office, and the Alliance staff manually entered the information into the clinic stakeholder Rave study database. Because clinics could not be enrolled in OPEN prior to 2020, clinic-level data were captured in the clinic stakeholder Rave study database, specifically in a Rave folder attached to the first clinic stakeholder that was enrolled at that clinic.

When OPEN was updated to allow hierarchical enrollment by 2020, a clinic and a clinic stakeholder could be enrolled directly in OPEN and a corresponding Rave study database would be created to house the data associated with the patient and non-patient level of the hierarchy (i.e., clinics and clinic stakeholders); the Alliance SDMC then pushed enrollments accordingly to their respective Rave study database obviating the need for Alliance staff to manually enter the non-patient enrollment information into their corresponding Rave study database. However, substantial work needed to be done within the Alliance SDMC to take advantage of this new multilevel enrollment functionality. The effort needed to build the necessary infrastructure, document and test the functionality within the Alliance SDMC was extensive, and required cross-functional collaboration among Alliance IT personnel, Alliance systems, and Rave developers, and spanned nearly 6 months before the complementary functionality was ready for production.

To accommodate non-patient participant enrollment in OPEN, systems integration between OPEN and the Alliance Research Enrollment Application needed to be put in place. Specifically, to leverage the non-patient participant enrollment in Rave, we needed to update our Research Enrollment Application that is responsible for creating unique subject identifiers (IDs) and randomization assignments. Additionally, updates were made to the integration between our Research Enrollment Application and Rave to push data between the two systems. Updates were also made to downstream processes—specifically the process for creating analysis files that combine data from the outputs in the two source systems (Research Enrollment Application and Rave), resulting in a more analysis ready dataset. Additional data capture elements were added to the template Demography and Subject Enrollment forms capturing the patient to non-patient link (e.g., linking a patient to their clinic and surgeon) and subject type. In addition, some demographic fields were removed from the non-patient Demography form template designed for non-patient participants that were not applicable such as method of payment.

The following are the non-patient participant types: provider, caregiver, other professional discipline, and practice. Because non-patient participants needed a unique “subject ID” across all CCDR studies, we applied the following naming convention of the non-patient participants: (1) PRO1, …, PRO9999 to capture providers, (2) CAR1, …, CAR9999 to capture caregivers, (3) OPD1, …, OPD9999 to capture other professional disciplines, and (4) PRA1, …, PRA9999 to capture practices. The hierarchical relationship among the patient and non-patient participants (e.g., patient is nested within a provider, who is nested within a practice) is not immediately established at the point that a non-patient participant is enrolled in OPEN; for example, when a provider is enrolled, it is not immediately clear which patients will subsequently enroll in the study under that provider. However, the link between the patient and non-patient participant is captured within OPEN and pushed to our Research Enrollment Application and to Rave on a weekly basis and is displayed on the Patient Demography Form making it readily available for use in data editing within Rave or near real time reporting. More dialogue on the implications of not having the link between the patient and non-patients established at the point of enrollment is made in section *Multilevel Data Monitoring*.

See Fig. 1b for an illustration of how data contained in our Research Enrollment Application and Rave source systems are combined and distilled into consumable analysis files. Our Research Enrollment Application collects the different enrollment types as unique trials as it was determined this provided the most long-term flexibility as well as the ability to permit different protocol schemas, eligibility criteria, and subject identifiers at the participant-type level. Designing a data collection system within Rave can get complicated when the participant types are particularly unique in their data collection needs; thus, there are advantages in designing the Rave databases unique to the participant types. While this differs from the CTSU-designed Rave access module, Alliance designed this multilevel data collection whereby enrollment information is pushed to Rave using the patient and non-patient designations. Including a trial for each participant type in our Research Enrollment Application allows for flexibility if this would need to change or if new participant types are added.

Study A231901CD, which was activated in January 2021, was the first CCDR trial in our portfolio to leverage the multilevel enrollment enhancements made to OPEN. In A231901CD, there is a patient-level randomization component such that patients are randomly assigned to receive a URL corresponding to an enhanced or standard web-based intervention. The approach to ensure that the patient receives the randomly assigned URL immediately after enrollment was detailed in the protocol and is described diagrammatically in Fig. 1c. Although OPEN and our Research Enrollment Application are used for patient enrollment and randomization, the local research study team for A231901CD is responsible for delivering the URL for the enhanced versus standard web-based platform via email *directly* to the patient. To accomplish this, the study chair or designee on the study research team, who is rostered as a special member within the Alliance, is given limited access to the *CTSU: Cancer Trials Support Unit OPEN* website, which will contain a study-specific cumulative report. The cumulative report contains four key data fields (patient’s Rave ID, enrollment date, email address, and the intervention to which the patient was randomly assigned) that are contained on the OPEN enrollment form. The study chair or designee logs in to OPEN and navigates to the reports tab and the CSV file (cumulative report) is downloaded/saved. So that the study chair (or designee) does not need to log in daily to OPEN, CRISP notifications are available so that the study chair (or designee) is notified when there is an enrollment to the study; the notification only provides site and patient Rave ID, enrollment date, site identifier, and treating investigator. The study research team then emails the patient the correct URL corresponding to the intervention assigned within the post-randomization time-period specified in the protocol.

While considerable time and effort was required to leverage the new multilevel enrollment functionality in OPEN, the enhancements made to support CCDR trials were welcomed and obviated the need to develop workarounds to collect information about health care providers/clinicians, caregivers, and health care organizations; furthermore, the enhancements still largely afford the desired flexibility that study teams oftentimes need when conducting CCDR trials in real-world settings.

### Quantitative and qualitative data capture

There were several unanticipated challenges associated with the myriad data collection methods for quantitative and qualitative data with our CCDR trials. Herein, we focus on four of the challenges and describe in detail each strategy applied to address the challenges. These four challenges encompass data capture methods from Qualtrics survey data, audio recordings, data obtained from a call center, and implementation data.

1. Application interface to load qualtrics survey data into rave

**Fig. 2:**
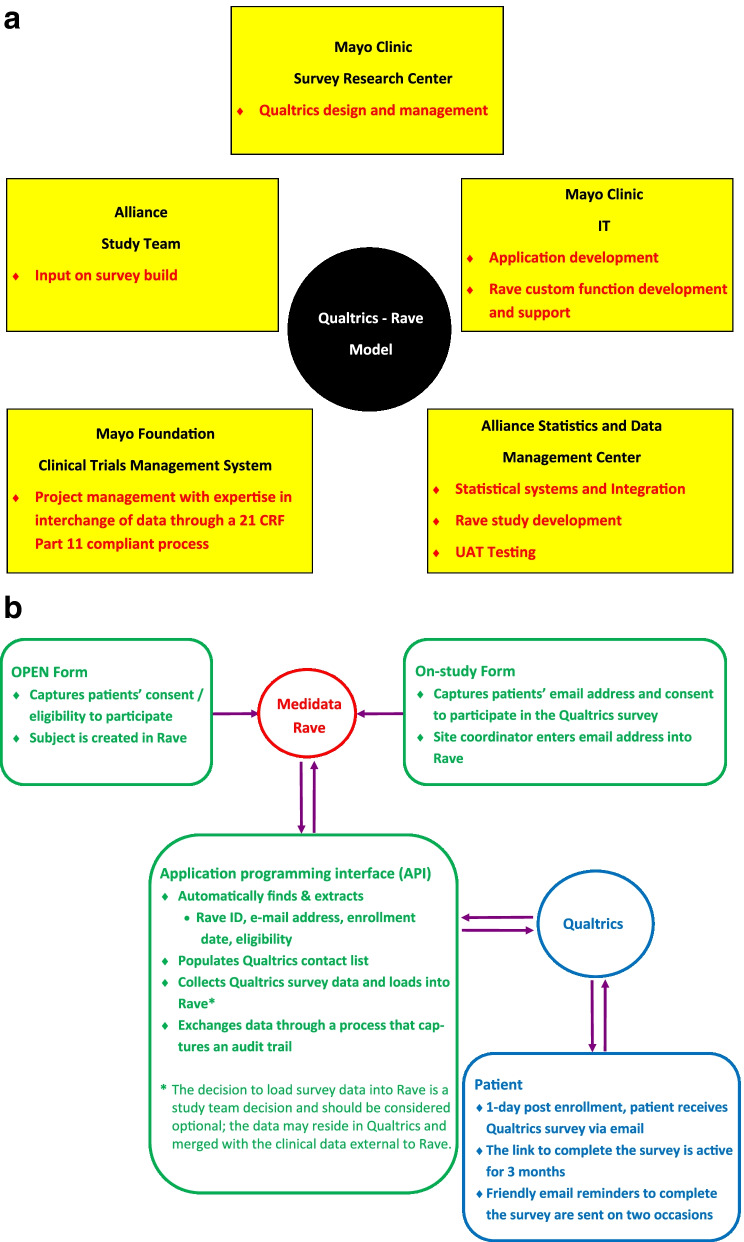
**a** Stakeholders assembled to develop the qualtrics—Rave mode. **b** Application interface to load qualtrics survey data into Rave

In trial A231701CD, it was thoughtfully decided to capture patient-reported outcomes (PROs) via a Qualtrics survey, as opposed to using a paper booklet or the electronic PRO (ePRO) data capture system. Although ePRO interfaces directly with Rave, the system requires patients to register and sign-in, compared with simply clicking a link to the Qualtrics survey; in study A231701CD with a single survey and a study population enriched with lower income participants, the study team felt that the ePRO registration step would be a considerable barrier. The challenge then was to provide a stable, configurable data interface, to provide for interchange of data between the Alliance instance of Medidata Rave and the Mayo Clinic instance of Qualtrics. To that end, we assembled a team of cross-functional stakeholders at Mayo Clinic (Fig. 2a) to develop the novel infrastructure to deliver the Qualtrics survey and exchange data between Rave and Qualtrics.

Mayo Clinic IT was responsible for developing an application interface (API). The API developed provided a configurable method and custom function that allowed Rave to instantiate a research subject in Qualtrics, with a unique identifying link, at the time that the subject was created in Rave (Fig. 2b). The strategy also included a mechanism through an application interface to link and load the PRO data in a Qualtrics survey into a corresponding eCRF in Rave. Importantly, the API was configurable for future trials, automated (running without manual intervention), and exchanged data through a process that captured an audit trail, which included the start and end date/time that the Qualtrics survey was completed. End user authentication data and collection of specific data from Qualtrics were loaded into Rave. Although Qualtrics survey data was a different data collection modality from Rave, we were able to maintain these survey data in Rave.

In this approach, the patients’ surgical consultation date, email address, and consent to participate in the Qualtrics survey was captured on the On-study form in Rave as opposed to on the OPEN form. Consequently, the site-coordinator’s timely recording of this salient information was needed because this information was used to trigger the Qualtrics survey to be emailed directly to the patient the day after the surgical consultation.

2. Data capture of audio recordings into rave

In trial A231701CD, the surgical consultation between the patients and their surgeon were audio recorded and the corresponding audio files were sent by the site to the study research team via a secure file transfer. All audio recordings were transcribed in full by the study research team. Each transcript file was uploaded as a PDF document into Rave and linked to the respective patient using a patient identifier.

Each transcript file was reviewed and coded by the study research team to obtain salient quantitative information from the audio recordings needed in data analysis. To maintain the blinded review and coding of the audio recordings (i.e., to ensure that the transcriber on the study research team did not know to which intervention arm the patient was assigned), the Alliance SDMC randomly selected patients in batches and sent a list of Rave patient identifiers to the study team. A231701CD was a stepped-wedge design where all clinics began in the usual care arm and then transitioned at randomized times to the intervention arm. To accommodate such a design and ensure blinded coding, a weighted algorithm was applied to obtain a desired balance of audio recordings between the two arms to send to the study research team. The study research team recorded the quantitative and salient information needed in data analysis, including key qualitative data, in an Excel spreadsheet. In parallel, the Alliance SDMC developed a corresponding case report form to receive periodic batch uploads of these data into Rave.

3. Data capture via a call center

In trial A231602CD, a lengthy patient telephone survey was administered by a call center and was required to be completed within 8 weeks of patient enrollment. The survey took 1 h to administer, on average, often requiring more than one telephone call to complete all survey questions. The survey questions included conditional branching (or skip logic) and parent/child-like questions such that some questions were not required based on answers to earlier questions. In Rave, the survey also included our standard comment functionality of a log line at the bottom of the survey. The study experienced rapid accrual, averaging 45 patients per month, and achieved its target accrual of 500 patients in less than a year. At the time of study activation, quality control measures were put in place to ensure interoperability between the patients’ responses and the corresponding data entered on the Rave case report form. To that end, the following process was implemented at the time of study activation. The administrator conducting the interview at the call center entered the data from the phone call onto a Microsoft Word document *during* the patient interview; this document served as the source documentation. The administrator later entered the data into Rave from that source document. Rave edit checks were built to ensure data conformity and that data for all expected fields were entered. The challenge that the call center faced was that for such a lengthy survey, the Rave edit checks were triggering numerous queries that disrupted the workflow. Stated differently, managing the queries and data entry became onerous within the context of a high-accruing and fluid study.

As this was our first CCDR trial using a call center, we experimented with three approaches to address this unanticipated challenge throughout the course of the study. First, instead of recording the data on source documentation, the interviewer entered the data directly into Rave during the patient interview. However, the edit checks were triggering queries in real time during the interview, hampering the ability to conduct the interview, perform Rave data entry, and manage the queries being triggered. Particularly, our original survey built in Rave did not include response selections of “patient does not know,” “not asked,” and “patient prefers not to answer” for each survey question; the administrator while conducting the interview would indicate these responses on the comment log at the bottom of the survey in Rave. Because of how the comment log was set up in Rave, when the administrator indicated a survey question with a comment of, say, “patient prefers not to answer,” the administrator needed to save the case report form in Rave each time to complete a log addition. Saving the form in Rave to complete each log addition caused all edit checks on the case report form to populate, including those edit checks that ensured data for all expected fields were entered inclusive of those survey questions that had not been asked yet at that point in the interview. As our second approach and in collaboration with the Alliance SDMC, the edit checks were then removed. While such an approach streamlined the interview and Rave data entry process, it quickly became apparent that data quality was at risk of being sacrificed. Our third strategy and the strategy that was adopted for the remainder of the study was to record the data on the source documentation during the interview and then after the interview the interviewer entered the data from the source documentation into Rave without edit checks; additionally, we revised parts 1–3 of the survey in Rave (we were not permitted to edit the curated survey questions in part 4) to include the response selections of “patient does not know,” “not asked,” and “patient prefers not to answer” to each survey question due to the frequent need to record such responses. A designated, albeit independent staff member at the call center would perform a review for accuracy 1–2 weeks after data entry into Rave. If there were discrepancies between the source document and Rave, the interviewer was asked to correct them. Further, the source document was saved on a secure server at the call center.

4. Implementation data collection

The interventions studied in CCDR are prone to differ in their implementation in different settings. Central to CCDR is gathering information on implementation of the interventions to advance our understanding of organizational, systems, and policy factors that affect implementation and to inform necessary adaptation or modifications that may need to be made to the interventions in real-world clinical settings. To that end, our CCDR trials conduct process evaluations (pre-, during-, and post-implementation) to obtain implementation outcomes via observational data, surveys, and interviews with enrolled patients and clinic stakeholders to assess the success of the intervention implementation. Collecting implementation outcomes, which are unique to CCDR, raised two unanticipated challenges with database development and data monitoring.

The interventions being studied in our trial portfolio to date generally consist of a tool used to inform patients about available treatments, along with potential benefits, risks and costs, during clinical encounters (A191402CD, A231701CD, and A231901CD), or to implement frailty screening and preoperative optimization for older patients (A231601CD; OPTI-Surg toolkit). The decision aids can only be effective if patients are able to access and review it. Similarly, the OPTI-Surg toolkit can only be effective if the toolkit can penetrate clinical practice. Therefore, it is important to monitor the reach of the decision aids and penetration of OPTI-Surg administration by tracking the screening (or participation) rate among eligible participants, defined as the number of participants administered the intervention divided by the total number of eligible participants. Further, it is important to also monitor limited demographics of those individuals who consent to the research study but decline the intervention to ensure that the interventions are not systemically discriminating against a demographic group as we are doing in A231701CD. However, because we cannot collect identifiable participant-level data from unconsented participants (more dialogue is provided on this topic in the discussion), we created study-specific Rave forms to capture monthly, aggregate-level practice data from eligible participants who do not consent to participate in the study (see Additional file 1: Appendix I for two examples of such Rave forms). Clinic staff complete these forms at the end of each month. Retrospective data capture in this manner can introduce inaccurate numbers or result in missing data; therefore, we needed to monitor these forms monthly for each clinic, reaching out to a clinic as needed, to ensure timely completion of these forms throughout the clinic’s participation in the trial.

To evaluate multilevel facilitators and barriers to implementation of the interventions, ethnographic methods, including direct observation of clinic routines, discrete survey questions, and interviews with patient and non-patient participants’ interviews are conducted. Information learned is incorporated to optimize the integration and future dissemination of the interventions. Oftentimes, data collected during the conduct of the trial are needed by the study team to adapt strategies for implementation during enrollment, to sample patient and non-patient participants for interviews, and to maximize interview quality. Because several of our CCDR trials are under the purview of the Alliance Data and Safety Monitoring Board (DSMB), study teams needed to formally request and receive approval for release of study data by the Alliance DSMB to ensure minimal impact to achieving the main study aims. This process underscored the need for study teams to plan well in advance (i.e., detailing the planned use of the data in the protocol, the specific data fields needed, the timing of when those data would be needed, and how to operationalize the process) to ensure data availability, adequate Alliance SDMC resources to operationalize the process, and sufficient time for the Alliance DSMB to review the data release requests, as well as to maintain fidelity to Alliance policy on data transfers to the study team during the conduct of the trial.

### Multilevel data monitoring

Multilevel data monitoring poses challenges for both the site coordinators and the Alliance SDMC. Trial A231901CD was the first CCDR trial to be activated that implemented the 2020 enhancements to OPEN to allow non-patient participants to be enrolled within OPEN. Although this enrollment feature was a welcomed enhancement for the Alliance, the hierarchical relationship among the patient and non-patient participants (e.g., patient is nested within a clinic stakeholder, who is nested within a clinic) is not immediately established at the point that a non-patient participant is enrolled in OPEN. Rather, a member at the site who performs the enrollment in OPEN is required to return to OPEN post-enrollment to manually link the non-patient participant to the patient; there is a “link to” field on the summary screen in OPEN. Because the timely linking of non-patient participants with the patient is needed for routine reporting purposes and communication with the participating sites, the Alliance SDMC is generating weekly monitoring reports to identify all enrollments that have not been manually linked by the clinical research professional (CRP) within 48 h of enrollment. The data manager within the Alliance SDMC then reaches out to the CRP in question to remind them to log into OPEN to establish the link between the patient and non-patient participants.

We have found that orienting site coordinators to each Rave study database, which corresponds to a level in the hierarchy (patient; non-patient participant), was needed as site coordinators were unfamiliar with multiple study databases for a single Alliance clinical trial. Further, site coordinators were not conditioned to monitor more than one study database within Rave, oftentimes neglecting to routinely monitor data from non-patient participants. With the 2020 enhancements to OPEN, we were able to leverage the standard navigation package in Rave that was set up and tailored for patient enrollment on our non-CCDR Alliance trials, which comprise > 95% of the trials conducted within the Alliance. Our standard navigation package creates target folder dates at the time of enrollment. This means that an overdue icon is immediately generated when a form is overdue, i.e., as soon as it passes the internal target folder date. Put another way, we can rely on Rave to inform the site that a particular form is overdue. Once the site coordinator logs into Rave they will see the overdue icon.

Prior to the 2020 enhancements to OPEN and as a cost- and time-saving alternative to customized calendaring for each CCDR trial, we judiciously decided to create monitoring reports external to Rave, which relied on a manually created target date folder for clinic stakeholder- and clinic-level data post enrollment. If we flag an overdue form for a non-patient participant (clinic stakeholder or clinic), we relayed that information to the SDMC data manager who in turn emailed the site coordinator to go into Rave and complete the form in question. While the advantages of the new multilevel enrollment feature in OPEN are clearly helpful, the Alliance SDMC still needs to perform some routine monitoring of clinic stakeholder- and clinic-level data. As previously mentioned, our participating sites are not accustomed to having > 1 study within Rave (patient; non-patient participants). The site needs to habitually log into each study database if they are to see the overdue icon. Therefore, we monitor these forms externally and relay to the data manager that a non-patient participant form is overdue; in turn the data manager reaches out to the site and reminds the site coordinator to log into the non-patient participant database and complete the form in question in a timely fashion. For example, if we wait 6 months to a year for the site to complete their clinic-level form that captures clinic-level characteristics, then we risk two different sets of site characteristics (one early; and one later) because site characteristics change and may change frequently. Also, the baseline forms for the clinic stakeholder participant (e.g., surgeon) need to be completed before he/she accrues a patient. Again, the timeliness of data capture is paramount, and external data monitoring at these other levels, which sites are not accustomed to (including the clinic stakeholders) is important for timely data submission.

## Discussion

CCDR is an emerging field. In this article, we detailed the innovative strategies that the Alliance SDMC applied to several unanticipated challenges in our CCDR trials related to multilevel enrollment; multilevel quantitative and qualitative data capture, including nontraditional data capture mechanisms; and multilevel data monitoring. Notably, the strategies adopted required thoughtful and sustained communication between the Alliance SDMC, research study teams, NCI, and participating sites. While we have accumulated considerable experience in this space, there remain several points of consideration and gaps that need to be addressed in database development and implementation for national, multi-site CCDR trials.

First, because the interventions being evaluated in CCDR trials tend to be necessarily implemented or delivered at the practice level, the designs are often characterized by the randomization of centers to interventions and patients are typically identified for participation after random allocation of the clusters; therefore, there is potential concern with sampling bias associated with CCDR trials [5, 6]. Once a site is informed of the study arm it was randomly assigned, the site is asked to enroll a targeted number of eligible patients and to implement the assigned intervention. Prior opinions of the different interventions and beliefs about their effectiveness may influence clinic staff and patients regarding enrollment decisions. At a minimum, we collect if a patient declines to provide consent to calculate the participation rate. However, in an attempt to address sampling bias directly, a monthly clinical practice data case report form in Rave was created (examples provided in Additional file 1: Appendix I). Within a single month/year, the practice is required per protocol to record in Rave the number of eligible patients offered participation in the study and the number of new patients seen in the clinic. However, we may also want information from nonconsenting patients to assess whether certain patients are not being represented. If patients’ decline to participate in a research activity, the site coordinator may record limited information about them including age, race, ethnicity, and reason for declining. These data points could be collected in an aggregate fashion within a site so that the study team may assess whether consented participants are categorically different than nonconsenting participants. It is unclear, however, how best to operationalize such a data collection strategy. Alternatively, given the limited amount of information that is collected, a waiver of informed consent could be considered for these subjects or limit the data collection to non-identifiable data with Institutional Review Board approval. In any case, to minimize sampling bias, remedial measures could be adopted during trial conduct based on monitoring such data in a meaningful manner.

A related point with respect to CCDR designs that employ randomization of centers to interventions is routine monitoring of accrual and important demographic and baseline characteristics to ensure that the trial is on track to achieving the protocol-defined targets of patient and non-patient enrollment assumed in the study design. The study’s pre-specified hypotheses may be tethered to achieving a targeted percentage of patients enrolled to certain demographics. For example, trial A191402CD aimed to produce significant generalizable inferences about the best way to improve prostate cancer decisions for minority men, particularly Native American and African American (AA) men [7]. Therefore, the study needed to over sample minority men in sufficient numbers to make robust inferences about their effects in these subgroups. The results from stepped-wedge CRTs are known to be highly sensitive to slight deviations in assumptions, including the number of patients enrolled per clinic per wave, on average [8]. Furthermore, for any given average number of patients per cluster in a CRT, statistical power will decrease as variability of cluster size increases [9]. Monitoring multi-level accrual in real time allows the study team to identify site-level barriers to accrual at each level of the hierarchy and within pre-specified, important subgroups so that measures can be taken to remove or mitigate those barriers. To facilitate the monitoring of multi-level enrollment, we designed the Rave databases to be unique to the participant types such that enrollment information was pushed to Rave using the patient and non-patient designations. Such a solution affords flexibility if new participant types are later added.

The aim of such routine monitoring is to identify and remove barriers to accrual and to maximize participation. Monitoring of demographics and key baseline characteristics to evaluate, say, the comparability of the study arms during the conduct of the trial would be the role of the DSMB; in other words, the study team, outside of the Alliance SDMC, should not have visibility to study-arm information on demographics and baseline characteristics or other sensitive trial information during the conduct of the trial. Further, to maintain the integrity of clinic-level randomization and the study design, both participants and study staff responsible for recruitment and enrollment should be kept blinded to the intervention arm that the clinic was allocated to when possible. Finally, transparent, and objective criteria for patient and non-patient recruitment and enrollment should be pre-specified prior to clinic-level randomization.

Second, if a level of the multilevel enrollment hierarchy elects to withdraw participation from the trial, there will likely be implications at a lower level of the hierarchy. For instance, if a clinic withdraws participation from the trial, what does that mean for an enrolled clinic stakeholder and patient at that clinic who are still in follow-up and a part of data collection per the study calendar? Or suppose the clinic seeks to withdraw from a component of the trial (e.g., clinic-level data collection) but would like to continue to accrue patients to the study. In our trials, we always allow participating patients to withdraw consent from any or all components of the study (e.g., patient-reported outcome data collection), and the reason(s) for withdrawal is (are) documented on a Rave case report form. It is crucial to also capture the reasons for non-patient participant withdrawal in Rave. For instance, clinics have withdrawn participation from our CRTs prior to accruing any patients to the study and a clinic stakeholder withdrew from the study because they left the clinic to pursue another employment opportunity elsewhere. Without a clinic- or clinic-stakeholder-level withdrawal of consent form, one is unable to systematically capture the reasons for withdrawal in the Rave study database in a standardized fashion. Further, allowing non-patient participants to continue in any aspects of the study, including data collection associated with the non-patient participant or the non-patient participant’s patient(s), may mitigate the instances of complete discontinuation from the study and the downstream implications associated with complete study withdrawal. Formally capturing the reasons for withdrawal from a component of the study or the entire study in Rave, as we customarily do with patients, can provide the needed context to evaluate whether any bias has been introduced as a result, which may in turn inform the design of subsequent trials to reduce such undesirable occurrences.

A third point of consideration is recognizing the value of DSMB oversight for a CCDR trial, which often has a complex design and challenging data collection needs. Alliance CCDR trials within the current portfolio are testing non-pharmacologic interventions with no anticipated safety concerns, and most do not have early stopping rules for efficacy or lack of efficacy. In all but one CCDR trial (A231602CD; the single observational trial), we leveraged the utility and expertise from the Alliance DSMB to facilitate multilevel data monitoring; to review the progress of multilevel accrual against protocol targets, including the review of patient and non-patient participant withdrawal rates; to review the quality of trial implementation; and to make recommendations regarding the continuation, termination, or modification of the trial. For instance, despite stratifying clinic-level randomization based on the proportion of patients the clinic sees with certain characteristics that may have a powerful impact on the study’s outcomes (e.g., socioeconomically disadvantaged patients or AA men as in the case of A191402CD), study arm imbalance in key patient-level factors is likely to occur between practices. If substantial imbalance is observed, the DSMB can advise the study team on whether adjustment for such imbalances in the analysis are necessary. The Alliance DSMB reporting template needed to be tailored to CCDR trial reporting to accommodate multilevel accrual and data collection. In some cases, there was also a need to introduce and orient the Alliance DSMB to the nuances of the unconventional trial design. However, the study team’s effort to prepare the bi-annual Alliance DSMB reports, including thoughtfully presenting the data in a manner conducive to review, coupled with the feedback received by the DSMB has been paramount to maintaining trial integrity of our CCDR trials.

Lastly, consideration should be given to adapting the Rave system of rolling out electronic case report forms and data monitoring to the types of interventions in CCDR trials. Our existing Rave navigational and data monitoring systems were designed for cancer therapeutic trials where participants regularly return to the clinic for treatment and follow-up. Sites are not in regular contact with patients in CCDR trials by design, with surveys frequently completed remotely via ePRO or mailed-in paper booklets, and thus, there are not opportunities for using trigger questions to roll out forms. Even if a patient fails to remotely submit their survey within the study window, we oftentimes still need to collect long-term follow-up information via medical chart reviews and, therefore, need follow-up forms to roll out. At times, we may also need to collect adverse events (AEs) related to the intervention, as in the case of the administration of the OPTI-Surg Toolkit (A231601CD). We also collect survival status on non-patient participants in study A231901CD (i.e., providers) even though it is not required per protocol because the corresponding form is needed for navigational purposes within our current Rave system. A simplified Rave navigational and data monitoring system tailored to the types of interventions in CCDR studies can reduce confusion and aid in high-quality data capture.

The strategies we applied were in response to unanticipated challenges we encountered and, therefore, longer term and more thoughtful and robust strategies may be desired. For example, health disparities and the digital divide [10] can no longer be an ancillary thought when designing clinical trials. We know that electronic capture of PROs in NCI clinical trial networks is a major initiative and, consequently, ePRO, which is fully integrated with Rave has been adopted and prioritized within the Alliance. However, the A231701CD study team chose to capture PROs via a Qualtrics survey because it was felt that the ePRO registration step would be a considerable barrier to participation, particularly, among socioeconomic disadvantaged participants. While studies are underway to address suboptimal ePRO uptake (see, for example, A221805-SI1, which is testing an educational resource), enhancements made directly to the mobile app that collects data from patient diaries and responses to questionnaires and transfers the data to the Medidata Clinical Cloud could be sought in collaboration with Rave developers to obviate the desire by study teams to use other data collection modalities such as Qualtrics. Additionally, the OPEN enrollment form could be modified to collect the zip code+4 for all patient participants in NCI clinical trial networks, rather than the current 5-digit zip code. The zip code+4 can be used to determine a valid area deprivation index [11], and this information can assist with the identification and monitoring of socioeconomically disadvantaged participants.

## Conclusion

Due to the complexity of and unique points of consideration with CCDR trials, the Alliance SDMC needed to explore and apply innovative strategies to address the myriad unanticipated issues and challenges with database development, centralized data management, and multilevel data monitoring of CCDR trials using the clinical trial data management system Rave. Although the challenges we faced were surmountable, our shared experience can be used to inform and assist others that are conducting such trials, as well as to foster collaboration to collectively build on past successes and improve on past failures.

ClinicalTrials.gov Identifier: NCT03103321 (Alliance A191402CD); NCT03857620 (Alliance A231601CD), NCT03766009 (Alliance A231701CD), and NCT04549571 (Alliance A231901CD)

## Supplementary Information

**Additional file 1: Appendix I.** Rave Case Report Forms to Capture Monthly Practice Data for Trials A231601CD and A231901CD.

## Data Availability

Not applicable.
