# Supplementary material for: Successes and lessons learned in database development for national multi-site cancer care delivery research trials: the Alliance for Clinical Trials in Oncology experience
Source: Trials. 2022 Aug 9;23:645. doi: 10.1186/s13063-022-06536-x (PMC9364584; doi:10.1186/s13063-022-06536-x)
Supplement: Supplementary file 1 — Additional file 1: Appendix I. Rave Case Report Forms to Capture Monthly Practice Data for Trials A231601CD and A231901CD. [file 13063_2022_6536_MOESM1_ESM.pdf]

APPENDIX 1

Rave Case Report Forms to Capture Monthly Practice Data for Trials A231601CD and A231901CD

Alliance Cancer Care Delivery Research Study A231601CD

Alliance for Clinical Trials in Oncology

Protocol Number: **A231601CDSite**

Subject ID: ______________________

Institution (Inst. Number): ______________________

Monthly Practice Data – Referral Data

Instructions: Report in this table, eligible patients who do not consent to participate in questionnaire completion (patients who consent to participate in questionnaire completion should be registered through OPEN). Each eligible patient reported in this table should only be included within a single Month/Year according to the Month/Year in which he/she was first identified for study entry.

Month/year of study entry (MMM/yyyy)	Number of eligible patients	Number of eligible patients administered EFS	Number of eligible patients given referral(s) based on EFS	Number of eligible patients following through with referral(s)
_ _ _/_ _ _ _	___	___	___	___

Add additional log lines for each month the site is actively participating in the study.

ALLIANCE FOR CLINICAL TRIALS IN ONCOLOGY
Protocol Number: A231901CD-NP
Participant ID: ______________________
Institution (Inst. Number): ______________________

Monthly Practice Data (Practice Only)

Month/year of Study Entry (MMM/yyyy)	Number of New Breast Cancer Patients Seen in the Clinic	Number of Patients Offered Participation in the Study
_ _ _/_ _ _ _	___ ___ ___	___ ___ ___

Add additional log lines <u>for each month</u> the site is actively participating in the study.
